# SARS-CoV-2 Seroprevalence among Canadian Blood Donors: The Advance of Omicron

**DOI:** 10.3390/v14112336

**Published:** 2022-10-25

**Authors:** Sheila F. O’Brien, Niamh Caffrey, Qi-Long Yi, Chantale Pambrun, Steven J. Drews

**Affiliations:** 1 Epidemiology and Surveillance, Canadian Blood Services, Ottawa, ON K1G 4J5, Canada; 2 School of Epidemiology and Public Health, University of Ottawa, Ottawa, ON K1H 8L6, Canada; 3 Medical Affairs & Innovation, Canadian Blood Services, Ottawa, ON K1G 4J5, Canada; 4 Department of Pathology & Laboratory Medicine, University of Ottawa, Ottawa, ON K1H 8L6, Canada; 5 Medical Microbiology, Canadian Blood Services, Edmonton, AB T6G 2R8, Canada; 6 Department of Laboratory Medicine & Pathology, Division of Diagnostic and Applied Microbiology, University of Alberta, Edmonton, AB T6G 2R3, Canada

**Keywords:** SARS-CoV-2, seroprevalence, Canada, blood donors, Omicron

## Abstract

With the emergence of the SARS-CoV-2 Omicron variant in late 2021, Canadian public health case/contact testing was scaled back due to high infection rates with milder symptoms in a highly vaccinated population. We monitored the seroprevalence of SARS-CoV-2 nucleocapsid (anti-N) and spike protein (anti-S) antibodies in blood donors across Canada from September 2021 to June 2022 in 202,123 randomly selected samples. Multivariable logistic regression of anti-N positivity with month, age, sex, racialization, region, material and social deprivation (based on postal code) identified as independent predictors. Piece-wise logistic regression analysed the association between anti-S concentration and month, and anti-N/anti-S positivity. Infection-related seroprevalence (anti-N positive) was 4.38% (95% CI: 3.96, 4.81) in September reaching 50.70% (50.15, 52.16) in June; nearly 100% were anti-S positive throughout. Anti-N positivity was associated with younger age, male sex, the Alberta and Prairies regions, greater material deprivation and less social deprivation (*p* < 0.001). Anti-S concentration was high initially (3306 U/mL, IQR 4280 U/mL), increased to (13,659 U/mL, IQR 28,224 U/mL) by June (*p* < 0.001), following the pattern of deployment of the third and fourth vaccine doses and was higher in those that were anti-N positive (*p* < 0.001). Despite already high vaccination-related seroprevalence, infection-related seroprevalence increased dramatically with the emergence of the Omicron SARS-CoV-2 variant.

## 1. Introduction

In Canada, the first case of the SARS-CoV-2 Omicron variant was reported on 28 November 2021. By mid-December 2021, it was the dominant variant [1]; the Delta variant had almost completely disappeared by late March 2022 [2]. S-gene target failure (SGTF) nucleic acid assay tests (NATs) tracked broad changes in S gene polymorphisms through NAT failure in several jurisdictions [3,4,5]. These polymorphisms in the SARS-CoV-2 Omicron S gene were not present with the Delta variant and were postulated to be linked to vaccine escape [3] facilitating the emergence of the Omicron variant in already vaccinated populations. By November 2021, approximately 80% of Canadians had received one dose of a Health Canada-approved COVID-19 vaccine; 75% had received a primary series. By 11 December 2021, about 9% had received a third dose [6].

Both because increasing infections overwhelmed public health testing facilities and because Omicron variant symptoms were less severe in healthy people [7], molecular diagnostic testing was scaled back nation-wide in December 2021 [8]. Numbers of intensive care unit (ICU) and mechanically ventilated cases remained low compared to previous SARS-CoV-2 peaks, while detection of SARS-CoV-2 in hospitalized patients was often incidental [9]. Testing policies varied by province but tended to prioritize healthcare workers and those at risk of severe disease. Provinces lifted prevention restrictions at varying timepoints over the spring of 2022, including the removal of masking requirements, social distancing guidelines, capacity restrictions, vaccination passports and mandatory quarantine for people who are infected [10].

With reduced public health testing, indicators of infection rates were derived from wastewater surveillance and seroprevalence studies. Wastewater surveillance is an early indicator available at sentinel sites in Canada which use variable methodologies, and it does not provide the proportion of the population infected [11]. Seroprevalence studies have a time delay of about 6 weeks until available but are the best indicator of the true proportion of infections. Since May 2020, Canadian Blood Services has monitored SARS-CoV-2 seroprevalence to inform public health policy [12,13]. With almost 500,000 samples tested to date and near national coverage, it is the largest seroprevalence study in Canada. Prior to the emergence of Omicron, we reported low infection-related seroprevalence in Canada, but very high vaccination-related seroprevalence in line with high adult vaccination rates [6,12,13]. To date, publications describe a rapid increase in seroprevalence with the Omicron variant in Europe [14,15], the US [16], South Africa [17] and Canada [18] but do not extend beyond March 2022 except one study from a region in northern Spain [19]. The surge in infections has continued over the spring and summer of 2022 fueled by Omicron subvariants. In this study, we aimed to evaluate SARS-CoV-2 seroprevalence in Canadian blood donors from September 2021 to June 2022 as the Omicron wave of infection advanced.

## 2. Materials and Methods

### 2.1. Study Design and Population

Prior to donating blood, all donors complete a screening questionnaire to ensure they are in good health and not at risk for blood transmissible infections. Throughout the pandemic, measures were in place at collection sites to reduce the risk of transmission of SARS-CoV-2 among staff and donors. This includes a two-week deferral from donating blood for donors who had close contact with someone infected, or if they have had an infection. If hospitalization was required, a three-week deferral is in place. All donors are checked to ensure they are afebrile prior to donating.

Canadian Blood Services collects blood donations from 9 of 10 provinces (excluding Québec and the northern territories), in all larger cities and most smaller urban areas. With each donation, an extra ‘retention’ plasma sample is collected in case additional testing is required. In most cases (approximately 80%), these extra samples are not required for the release of blood products and were available for serological testing for this study. Samples were randomly selected from the last two weeks of each month from September 2021 to December 2021 and from all weeks from 1 January 2022 to 30 June 2022. This study was approved by the Canadian Blood Services Research Ethics Board.

### 2.2. Serologic Testing

Retention plasma samples were aliquoted and stored frozen at −20 °C or colder until processing at the Canadian Blood Services laboratory in Ottawa, Ontario. Two assays were used to test each sample: the Roche Elecsys^®^ Anti-SARS-CoV-2 spike semi-quantitative immunoassay (Roche Diagnostics International Ltd., Rotkreuz, Switzerland) which measures total antibodies (including IgA, IgM, and IgG) to the SARS-CoV-2 S1/receptor-binding domain (RBD) of the spike protein (anti-S) and the Roche Elecsys^®^ Anti-SARS-CoV-2 qualitative immunoassay (Roche Diagnostics International Ltd., Rotkreuz, Switzerland) which measures total antibodies (including IgA, IgM, and IgG) to SARS-CoV-2 recombinant protein, nucleocapsid antigen (anti-N). All samples were tested for anti-spike protein at a dilution of 1:400, and if negative were re-tested neat. Manufacturer estimates of Roche assay sensitivities and specificities were as follows: at a concentration of ≥0.8 U/mL, the anti-S assay was assumed to have a sensitivity of 98.8% at ≥14 days post PCR confirmation and a specificity of 99.97% [20]. At a sample-to-cut-off ratio of ≥ 1.0, the anti-N assay was assumed to have a sensitivity of 99.5% at ≥14 days post PCR confirmation and specificity of 99.8% [21]. Both anti-nucleocapsid and anti-spike protein antibodies are produced after natural infection, whereas only anti-spike antibodies are produced after spike-protein-based vaccination [22]. All Canadian approved vaccines are spike-protein-based [23].

### 2.3. Data Management and Statistical Analysis

Demographic variables were extracted from the Canadian Blood Services donor database and added to the test data including donation date, Forward Sortation Area (FSA) from the residential postal code, sex, age, and self-reported ethnicity. Donor date of birth and sex are verified by documentation provided by the donor at first registration. Postal code is verified by staff asking the donor at each donation. Geographical regions across Canada included British Columbia, Alberta, Prairies (Saskatchewan and Manitoba), Ontario, and the Atlantic region (New Brunswick, Nova Scotia, Prince Edward Island, and Newfoundland & Labrador). During donor screening donors self-identified in an optional question as White, Asian, Indigenous, Arabic, Black, South Asian, Latin-American, or Other. These were regrouped a priori as either “white” (the majority of donors) or “racialized groups” because the proportions in various non-white ethnicities were small. Socioeconomic status was estimated using the Pampalon Material and Social Deprivation Indices (MSDI) [24,25]. Material deprivation is associated with insecure job situation, insufficient income, and low education, while social deprivation refers to a fragile social network, characterized by living alone, being a single parent, or being separated, divorced, or widowed. MSDI was derived from 2016 Statistics Canada census, aggregated from postal codes to the dissemination area level (the smallest geographic unit available in the Canadian census, considering 400–700 persons) and were categorized into quintiles; from least deprived (1) to most deprived (5). Donors were categorized into 4 different age groups: 17–29, 30–39, 40–59, and 60+ years old. For anti-spike concentration analyses, the last age group was further broken down to 60–69, and 70+ years old. Data were weighted by raking for the Forward Sortation Area (FSA), age group, and sex to make inference to the general population based on Statistics Canada data (catalogue # 98-400-X2016008). For FSAs with few donors, several adjacent FSAs were combined to include at least 500 donors. In cases where no FSA was recorded or if not in a province where blood is collected (0.2% of samples), weighting was based on FSA of the blood centre. A heat map of anti-N seroprevalence was prepared using Statistics Canada economic regions which are standardized groupings of census divisions (catalogue # 92-195-X).

The weighted data were adjusted for sensitivity and specificity of the assay using the Rogan–Gladen equation [26]. The seroprevalence was calculated as the number of positive samples divided by all samples tested. The Exact method was used to estimate 95% confidence intervals (CI). SARS-CoV-2 seroprevalence was stratified by region, sex, age group, self-reported ethnicity (racialization), and MSDI by month.

Logistic regression was used to evaluate associations and risk factors by month for natural infection (anti-N positive). It is possible for one donor to be represented by more than one donation during the study period. However, the spike and nucleocapsid serostatus of a donor can change over time therefore samples were considered independent for the purpose of these analyses. Analyses were conducted using Stata/MP 17 (Statacorp, College Station, TX, USA) and SAS version 9.4 (SAS Institute, Cary, NC, USA).

Anti-S concentration (U/mL) by month was evaluated through linear regression. A piecewise linear regression model was fitted in which the slope was permitted to vary at the approximate dates when concentrations began increasing or decreasing (breakpoint), with before and after each breakpoint, sex, and anti-N positive as predictors. Separate linear regression models were used for each age group.

## 3. Results

Between 1 September 2021 and 30 June 2022, 230,123 retention samples were screened for SARS-CoV-2 anti-S and anti-N (Appendix A Table A1). Nearly all donors were anti-S positive throughout this period, while anti-N positivity remained low from September at 4.38% (95% CI: 3.96%, 4.81%) to immediately prior to an increase in Omicron cases in November at 5.04% (95% CI: 4.58%, 5.50%), then increased 50.7% (95% CI: 50.15%, 51.26%) by June. The proportions of donors were rather similar to the general population for age, sex and region, but were lower for racialization (18.3% of donors vs. 30% of the general population, see Appendix B Table A2).

**Figure 1:**
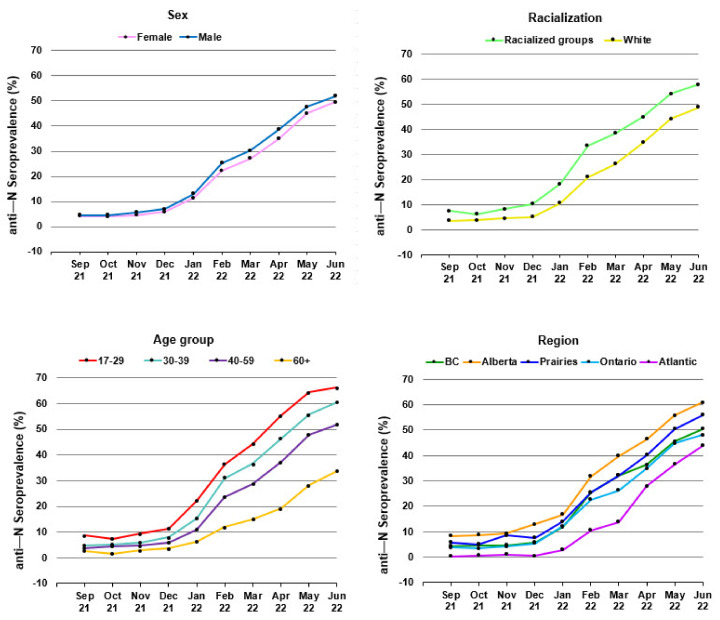
Anti—N seroprevalence from September 2021 - June 2022 by sex, racialization, age group, and region.

**Figure 2:**
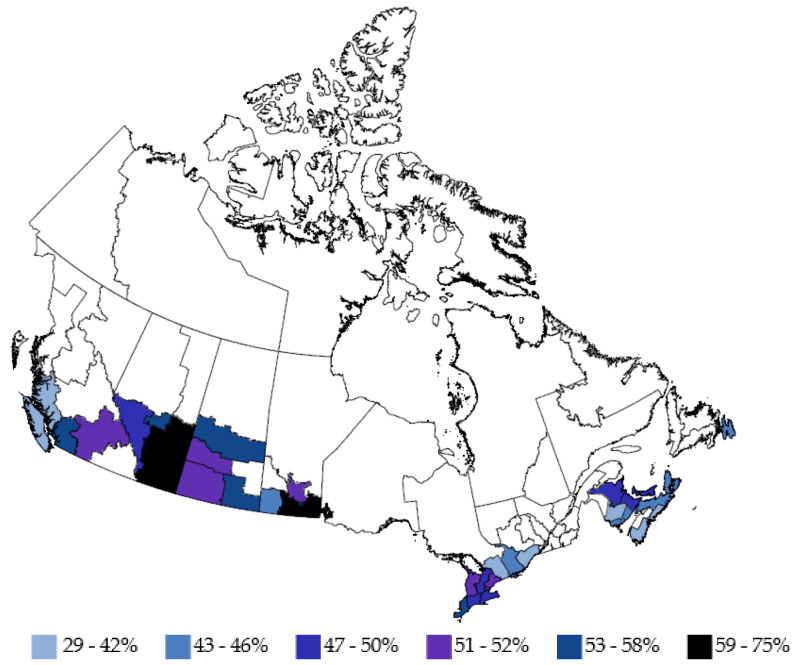
Anti-N seroprevalence in June 2022 by region. (White areas not tested/insufficient samples).

The percentage of donors who had anti-N positive results from September 2021 to June 2022 by sex, age group, racialization, and region are shown in Figure 1. A heat map of anti-N seroprevalence by region in June 2022 is shown in Figure 2. In multivariable logistic regression, anti-N positivity was significantly greater in males, younger donors, racialized donors, those living in the Alberta and Prairies regions and those living in more materially deprived neighbourhoods as well as those in less socially deprived neighbourhoods (See Appendix C and Appendix D, Table A3 and Table A4).

**Figure 3:**
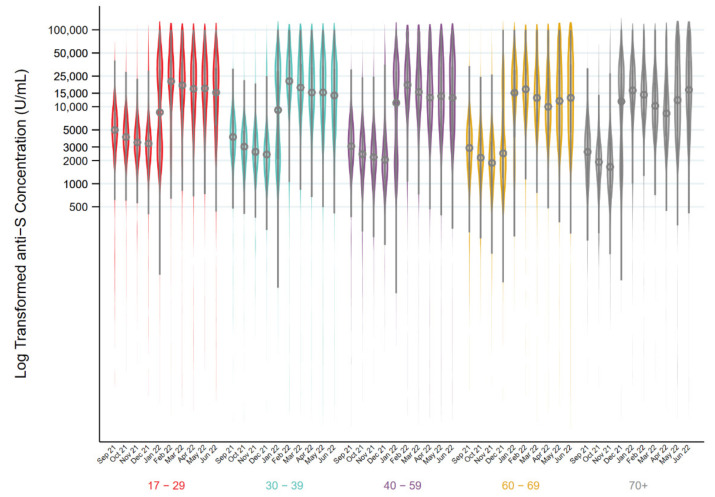
Distributions of log transformed Spike antibody concentration results (U/mL) (circle represents the median and the bar represents the interquartile range) in donations from September 2021 to June 2022 stratified by age group.

**Figure 4:**
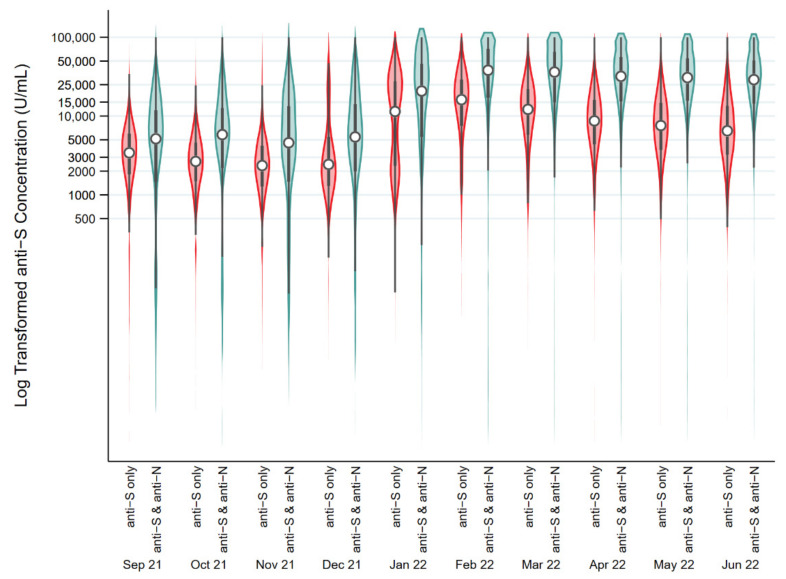
Distributions of log transformed Spike antibody concentration results (U/mL) (circle represents the median and the bar represents the interquartile range) in donations from September 2021 to June 2022 stratified by donors who were anti-S positive only or those who both were anti-S and anti-N positive.

Figure 3 shows the kernel density plots of the distribution and median anti-S concentration by month in all anti-S positive donations by age group. The piecewise linear regression models identified two breakpoints in the 17–29, 30–40, 40–59-year-olds with no change in slope up to mid-December, an increase up to the beginning of February, and a decrease afterwards (*p* < 0.001). In donors aged 60–69, there were three breakpoints with no change in slope until mid-December, an increase until late January, a decrease until the end of March, and an increase thereafter (*p* < 0.001). In donors 70 years and older, there were also three breakpoints, but earlier than the 60 to 69-year-olds (*p* < 0.001). There was no change in slope until late October, after which it increased until early January, decreased until the end of March, after which it increased (*p* < 0.001). Figure 4 shows the breakdown of anti-S concentrations by anti-N positive vs. anti-N negative donations. In all age groups, anti-S concentrations were elevated in donors who were also positive for anti-N (*p* < 0.0001). The overall median concentration of anti-S increased from September 2021 (3306 U/mL, interquartile range (IQR) 4280 U/mL) to June 2022 (13,659 U/mL, IQR 28,224 U/mL). Among anti-N positive donations, the median anti-S concentration increased from 5154 U/mL (IQR 10,440 U/mL) in September 2021 to 28,929 U/mL (IQR 36,253 U/mL) in June 2022. Among anti-N negative donations, anti-S concentrations were lower (*p* < 0.001) with 3248 U/mL, (IQR 4129 U/mL) in September 2021 up to 6388 U/mL in June 2022 (IQR 10,064 U/mL).

## 4. Discussion

In collaboration with the COVID-19 Immunity Task Force [27] formed by the Canadian Federal Government, we have monitored SARS-CoV-2 seroprevalence since May 2020. With almost 500,000 blood samples tested to date and near national coverage, ours is the largest seroprevalence study in Canada [12,13,28]. Here, we report a dramatic increase in infection-related seroprevalence in the first half of 2022 as the Omicron variant dominated.

We note that Omicron is a diverse cluster of subvariants and recombinants [29] with single nucleotide polymorphism antibody escape especially in regions of the RBD of BA.2.12.1, BA.4, and BA.5 [28,29,30,31]. Based on sequencing of specimens of convenience, by late March 2022, BA.2 had overtaken BA.1 with greater than 50% of sequenced specimens. Throughout June 2022, BA.5 replaced BA.2. BA.3 was negligible in sequenced Canadian specimens (≤0.5%) and disappeared after late June 2022. BA.4 remained at low levels (<15% sequenced by early July 2022) [9]. However, from a cell-mediated immune perspective, recent data suggest that T cell epitopes are conserved across Alpha, Beta, Gamma, Delta, and Omicron (BA.1, BA.2, and BA.3) [32,33], hence post-vaccination T cell immunity may be preserved against Omicron [34].

In Canada, adult vaccination rates with two doses were very high by the fall of 2021 due to completion of vaccine roll-out programs combined with requirements for proof of vaccination at most public venues. Hence, nearly all donors had spike protein antibodies. Vaccination strategies varied across jurisdictions with the use of mixed schedules and extended dosing intervals [35,36], which may have impacted the duration and strength of anti-SARS-CoV-2 humoral responses. The increases in spike antibody concentrations we observed are consistent with deployment of a third dose of vaccine in late 2021 which was prioritized initially to older individuals and high-risk groups (with some variability in policies by jurisdiction). We note that there is limited information to link anti-S as a correlate of protection. Antibody concentrations wane post-vaccination [13,37]. Although significant anti-S neutralization was reported 6 months after a second dose of BNT162b2 vaccine [38], in a test-negative case–control study, vaccine effectiveness against the Omicron variant decreased within a few months of a third dose of the BNT162b2 vaccine [39]. We identified higher anti-S concentrations among donations also positive for anti-N similar to Busch and colleagues [40] prior to Omicron and by Poon et al. in Hong Kong during the Omicron wave [41]. In a highly vaccinated population in Navarre Spain, anti-N positivity was associated with lower risk of Omicron infection (aOR = 0.08; 95% CI: 0.05–0.13) suggesting importance of hybrid immunity [19].

In spite of high spike concentrations throughout our study period, half of donors had evidence of infection by June, consistent with earlier reports of breakthrough infections [42,43]. This meant that with about 5% of donors anti-N positive prior to the emergence of Omicron, at least 45% of donors were infected over a six-month period. Furthermore, the true infection rate was likely higher because some infections may not have produced detectable anti-N [15] and waning anti-N has been reported 120 days post-infection [44]. Infections may have spread quickly in our population due to a variety of factors. Omicron subvariants carry a variety of adaptations that make them potentially more infectious [45]. A combination of milder illness with Omicron variants, high vaccine coverage and lessening public health restrictions may have facilitated greater community transmission by mildly ill infected individuals [46,47].

Published reports of Omicron wave seroprevalence in other countries cover time periods up to March 2022, although one smaller study in northern Spain reported results up to July [19]. For comparison, seroprevalence in Canada increased from 5% in November to 24% in February and 29% in March. This is similar to a smaller Canadian study from March 2022 [18]. It is also comparable to that reported in Helsinki, Finland at 28% in February/March [14] but lower than 58% in the US in February [16], 51% in Denmark in March [15], and 86% in South Africa in March [17]. In the US, higher seroprevalence early in the Omicron wave was additive to that of earlier time periods in the pandemic. In December, infection seroprevalence was reported to be 29% in a US national blood donor study [48], and 33% in a US general population report [16], compared with 6% in Canada. The surge of about 30% in the US is greater than roughly 18% in Canada up to February. In Denmark, the surge in the blood donor infection seroprevalence was much greater reaching 51% end of March [15], up from only 1.2% in November. Reasons for variable surges in infections after Omicron was present are multi-factorial. Some factors are similar across countries, such as the duration of the Omicron wave and populations with vaccination or other prior exposure. In each of these reporting countries, the first case of the Omicron variant was reported at about the same time as Canada (except in South Africa where it was earlier). While vaccination has been more protective against earlier variants, it provides less protection from infection with Omicron [49]. Hence, even in countries such as Denmark and Canada with high vaccination rates there was a large surge. In the US, vaccination uptake has been lower, but prior to the emergence of Omicron, the combination of vaccination and/or natural infection resulted in some humoral protection in the majority of people [40]. Omicron arrived at a time when most countries were reducing public health restrictions with little public appetite to re-instate. Hence, high transmissibility, vaccination resistance, and less social distancing have likely contributed [29,45].

Our results show that in Canada seroprevalence continued to increase up until June, reaching 50% of donors, but there was regional variability. The highest seroprevalence was seen in the Alberta and the Prairies regions as reported pre-Omicron [13]. Prior to the emergence of Omicron Atlantic Canada was largely spared from SARS-CoV-2 infection due to travel restrictions in and out of the so called “Atlantic bubble” and sparser population, but by June had reached 44%. Higher seroprevalence in younger age groups has persisted over the pandemic in Canada [13] and in other countries [19,50,51]. This has extended into the Omicron era and if anything has widened [14,15,16]. Higher prevalence of infection in racialized groups and those from neighbourhoods with lower socioeconomic status in our study are consistent with our previous report [13] and public health cases and mortality in Canada and other countries [50,52,53].

Key strengths of our study are the Canada-wide monthly sampling which permits tracking of seroprevalence over time by demographic groups and testing of both anti-N and anti-S to better differentiate likely natural infection from vaccination humoral response. There are also some important limitations. Vaccination and infection history are not collected. Random sampling of blood donations does not permit longitudinal analysis. Anti-S and anti-N concentrations do not measure neutralizing antibodies.

Throughout the pandemic, SARS-CoV-2 blood donor seroprevalence studies [12,13,28,48,50,51,54,55,56,57] have informed public health policy world-wide. Although some groups are excluded, notably children, those in poor health and those at risk of blood-borne infections, blood donors are a healthy population which permits wide-scale, timely, and cost effective seroprevalence estimates that are comparable with general population studies [40,58,59]. The unprecedented reliance on blood donor surveillance during the SARS-CoV-2 pandemic underscores the future potential for blood donors as a sentinel population in the context of epidemics. With the scale-back of public health PCR testing, seroprevalence studies provide more complete information on the burden of infection important for predictive modelling [60,61].

During an Omicron surge when public health surveillance options were decreasing in Canada, infection-related seroprevalence increased from 5% to 50%. This was despite pre-existing high spike antibody concentrations over the first half of 2022 as Omicron and its sub-variants dominated. Breakthrough infections over the Omicron wave have greatly increased hybrid immunity.

## Data Availability

Data may be made available upon request from Canadian Blood Services (contact person: Sheila O’Brien), subject to internal review, privacy legislation, data sharing agreements, and research ethics approval.

## Appendix A

**Table A1:** Number of samples tested and percentage of samples positive for anti-N and anti-S (weighted and adjusted for test sensitivity and specificity).

	Samples Tested	Anti-N Positive		Anti-S Positive	
	Number	%	95% CI	%	95% CI
September 2021	9363	4.38	3.96, 4.81	97.03	96.62, 97.44
October 2021	9627	4.26	3.85, 4.68	98.01	97.65, 98.36
November 2021	9018	5.04	4.58, 5.50	98.52	98.18, 98.86
December 2021	16,817	6.39	6.01, 6.76	98.58	98.34, 98.82
January 2022	32,505	12.12	11.76, 12.48	98.89	98.73, 99.06
February 2022	28,616	23.68	23.18, 24.18	99.60	99.45, 99.75
March 2022	26,057	28.71	28.16, 29.26	99.57	99.42, 99.73
April 2022	29,787	36.71	36.16, 37.26	99.74	99.60, 99.88
May 2022	31,764	46.32	45.77, 46.87	100.00	100.00, 100.00
June 2022	32,121	50.70	50.15, 51.26	100.00	100.00, 100.00

## Appendix B

**Table A2:** Demographic data (from donors donating between 1 September 2021 and 30 June 2022).

		Blood Donors	General Population (17 Years and Older) ^a,b^
Variable	Categories	(%)	(%)
Sex	Female	42.4	51.3
	Male	57.6	48.7
Age group	17–29	16.5	19.7
	30–39	17.9	17.0
	40–59	36.8	32.2
	60+	28.8	31.1
Racialization	White	81.7	70.0
	Racialized	18.3	30.0
Region	British Columbia	16.6	18.1
	Alberta	20.7	14.5
	Prairies	10.5	8.4
	Ontario	42.5	50.3
	Atlantic Canada	9.8	8.7
Material Deprivation Index	1 (least deprived)	29.8	19.9
	2	24.6	19.3
	3	20.5	19.9
	4	16.0	20.1
	5 (most deprived)	9.3	20.9
Social Deprivation Index	1 (least deprived)	21.2	23.2
	2	21.4	19.8
	3	20.2	19.5
	4	18.5	18.6
	5 (most deprived)	18.7	18.9

^a.^ Statistics Canada 2021 Census data. Census Profile, 2021 Census of Population. Limited to population 17 years and older and excluding the province and territories where blood donations were not collected (Quebec, Northwest territories, Yukon and Nunavut). From Table 98100026-eng.zip. Available online: https://www150.statcan.gc.ca/n1/tbl/csv/98100026-eng.zip, accessed on 31 August 2022. ^b.^ Statistics Canada 2016 Census Profile data. Data available from Table 98-401-X2016059_English_CSV_data. Available online: https://www12.statcan.gc.ca/census-recensement/2016/dp-pd/prof/details/page.cfm?Lang=E&Geo1=PR&Code1=01&Geo2=PR&Code2=01&SearchText=Canada&SearchType=Begins&SearchPR=01&B1=All&TABID=1&type=0, accessed on 31 August 2022.

## Appendix C

**Table A3:** The number of samples anti-N positive with the percentage and 95% confidence intervals in September 2021, November 2021, and June 2022 by demographic variables.

	September 21			November 21			June 22		
	Number Positive	%	95% CI	Number Positive	%	95% CI	Number Positive	%	95% CI
Sex									
Female	185	4.22	3.64, 4.80	201	4.48	3.87, 5.09	7289	49.61	48.85, 50.38
Male	253	4.56	3.94, 5.18	322	5.63	4.93, 6.32	8793	51.86	51.07, 52.65
Age group									
17–29	139	7.34	6.17, 8.52	167	7.66	6.43, 8.89	3211	65.39	64.23, 66.56
30–39	82	4.66	3.57, 5.75	100	6.16	4.91, 7.41	3266	59.01	57.68, 60.35
40–59	138	3.79	3.12, 4.47	159	4.73	3.97, 5.49	6340	51.99	51.06, 52.92
60+	79	2.78	2.13, 3.43	97	2.86	2.19, 3.53	3265	33.73	32.75, 34.71
Racialization ^a^									
White	109	7.61	6.24, 8.97	112	8.28	6.82, 9.74	3211	58.03	56.79, 59.27
Racialized	289	3.65	3.20, 4.10	372	4.56	4.05, 5.07	11,789	49.01	48.38, 49.65
Region									
British Columbia	74	4.23	3.24, 5.23	75	4.63	3.59, 5.68	2527	50.54	49.23, 51.86
Alberta	166	8.28	6.81, 9.74	195	9.25	7.67, 10.82	3771	60.86	59.45, 62.26
Prairies	75	5.73	4.08, 7.37	123	8.58	6.57, 10.59	1769	55.89	54.00, 57.78
Ontario	116	3.76	3.19, 4.32	116	4.04	3.45, 4.63	6887	48.14	47.37, 48.92
Atlantic	7	0.36	0.00, 0.87	14	1.01	0.25, 1.77	1128	43.87	42.02, 45.72
Material Deprivation Index ^b^									
1 (least deprived)	110	3.74	2.99, 4.49	119	4.54	3.69, 5.39	3999	48.53	47.42, 49.64
2	90	4.08	3.21, 4.95	92	4.01	3.11, 4.91	3349	49.46	48.26, 50.66
3	70	4.14	3.14, 5.15	89	4.78	3.67, 5.90	2920	49.72	48.43, 51.00
4	79	5.71	4.43, 6.99	96	6.31	4.97, 7.64	2278	51.95	50.51, 53.40
5 (most deprived)	29	4.02	2.57, 5.48	47	6.18	4.48, 7.89	1366	54.62	52.77, 56.48
Social Deprivation Index									
1 (least deprived)	90	4.65	3.66, 5.64	138	7.23	5.96, 8.49	3156	52.46	51.19, 53.73
2	82	4.90	3.88, 5.93	84	4.67	3.63, 5.70	2971	49.43	48.16, 50.71
3	71	3.91	2.94, 4.89	82	4.46	3.41, 5.51	2804	50.55	49.24, 51.87
4	75	4.16	3.15, 5.18	75	4.41	3.35, 5.48	2507	49.61	48.23, 50.98
5 (most deprived)	60	3.42	2.50, 4.34	64	3.61	2.65, 4.58	2474	48.56	47.18, 49.93
Total	438	4.38	3.96, 4.81	523	5.04	4.58, 5.50	16,082	50.70	50.15, 51.26

^a^. In September 2021, self-reported ethnicity data was missing for 802 samples of which 40 were positive (4.92%, 95% CI 3.37, 6.46). In November 2021, self-reported ethnicity data was missing for 752 samples, of which 39 were positive (3.35%, 95% CI 2.03, 4.68). In June 2022, self-reported ethnicity data was missing for 2219 samples, of which 1082 were positive (48.37%, 95% CI 46.24, 50.49). ^b^. In September 2021, postal codes were missing for 1031 samples of which 60 were positive (5.62%, 95% CI 4.16, 7.08). In November 2021, postal codes were missing for 1084 samples, of which 80 were positive (5.95%, 95% CI 4.51, 7.38). In June 2022, postal codes were missing for 4035 samples, of which 2170 were positive (54.29%, 95% CI 52.75, 55.82).

## Appendix D

**Table A4:** Multivariable logistic regression model with anti-N positive as the dependent variable.

Variable	Odds Ratio	95% CI
Month		
September 2021	Baseline	
October 2021	1.01	0.87, 1.17
November 2021	1.23 *	1.06, 1.43
December 2021	1.46 *	1.29, 1.66
January 2022	2.75 *	2.45, 3.08
February 2022	6.80 *	6.08, 7.61
March 2022	9.01 *	8.06, 10.08
April 2022	13.26 *	11.86, 14.82
May 2022	20.09 *	17.98, 22.45
June 2022	24.04 *	21.52, 26.86
Sex		
Female	Baseline	
Male	1.06 *	1.03, 1.08
Age group		
17–29	3.44 *	3.32, 3.56
30–39	2.70 *	2.61, 2.80
40–59	2.07 *	2.01, 2.13
60+	Baseline	
Racialization		
White	Baseline	
Racialized	1.23 *	1.19, 1.27
Material Deprivation Index		
1 (least deprived)	Baseline	
2	1.12 *	1.09, 1.16
3	1.19 *	1.15, 1.22
4	1.28 *	1.24, 1.33
5 (most deprived)	1.50 *	1.43, 1.56
Social Deprivation Index		
1 (least deprived)	Baseline	
2	0.92 *	0.89, 0.95
3	0.86 *	0.84, 0.90
4	0.83 *	0.80, 0.86
5 (most deprived)	0.77 *	0.74, 0.80
Region		
British Columbia	Baseline	
Alberta	1.46 *	1.41, 1.52
Prairies	1.11 *	1.06, 1.16
Ontario	0.86 *	0.83, 0.89
Atlantic	0.56 *	0.53, 0.58

* *p* < 0.001.
